# Toward the markerless and automatic analysis of kinematic features: A toolkit for gesture and movement research

**DOI:** 10.3758/s13428-018-1086-8

**Published:** 2018-08-24

**Authors:** James P. Trujillo, Julija Vaitonyte, Irina Simanova, Asli Özyürek

**Affiliations:** 1 Donders Institute for Brain, Cognition and Behaviour, Radboud University, Montessorilaan 3, B.01.25, 6525GR Nijmegen, The Netherlands; 2 Centre for Language Studies, Radboud University, Nijmegen, The Netherlands; 3 Max Planck Institute for Psycholinguistics, Nijmegen, The Netherlands

**Keywords:** Communication, Methodology, Motion tracking

## Abstract

Action, gesture, and sign represent unique aspects of human communication that use form and movement to convey meaning. Researchers typically use manual coding of video data to characterize naturalistic, meaningful movements at various levels of description, but the availability of markerless motion-tracking technology allows for quantification of the kinematic features of gestures or any meaningful human movement. We present a novel protocol for extracting a set of kinematic features from movements recorded with Microsoft Kinect. Our protocol captures spatial and temporal features, such as height, velocity, submovements/strokes, and holds. This approach is based on studies of communicative actions and gestures and attempts to capture features that are consistently implicated as important kinematic aspects of communication. We provide open-source code for the protocol, a description of how the features are calculated, a validation of these features as quantified by our protocol versus manual coders, and a discussion of how the protocol can be applied. The protocol effectively quantifies kinematic features that are important in the production (e.g., characterizing different contexts) as well as the comprehension (e.g., used by addressees to understand intent and semantics) of manual acts. The protocol can also be integrated with qualitative analysis, allowing fast and objective demarcation of movement units, providing accurate coding even of complex movements. This can be useful to clinicians, as well as to researchers studying multimodal communication or human–robot interactions. By making this protocol available, we hope to provide a tool that can be applied to understanding meaningful movement characteristics in human communication.

**Electronic supplementary material:**

The online version of this article (10.3758/s13428-018-1086-8) contains supplementary material, which is available to authorized users.

Human communication is intrinsically multimodal, consisting of not only speech but also visible communicative signals. Gesture, sign, and communicative actions (e.g., joint actions, demonstrations) are well-studied examples of communicative manual acts that can convey meaning in the presence or absence of co-occurring speech. A plethora of research in the last decade has shown that each of these modalities, although unique in certain ways, effectively utilizes movement and configuration to convey meaning and contribute to successful communication.

Among an array of visual bodily cues that people resort to when conveying meaning, gestures stand out as a unique attribute of the human communication system. A wealth of research has shown that gestures (we use the term “gestures” here to refer to movements of the hands and arms that are used to depict objects, ideas, events, and experiences; Kendon, 2004; McNeill, 1994) form an important aspect of communication. The study of gesture has opened a new window into human language, cognition, and interaction, (e.g., Kendon, 2004; McNeill, 1994; for a recent collection, see Church, Alibali, & Kelly, 2017), with important clinical applications, such as using the production and comprehension of pantomimes to assess disorders such as apraxia (Goldenberg, Hartmann, & Schlott, 2003; Gonzalez Rothi, Heilman, & Watson, 1985), autism spectrum disorder (Anzulewicz, Sobota, & Delafield-Butt, 2016), or Parkinson’s disease (Humphries, Holler, Crawford, Herrera, & Poliakoff, 2016).

Traditionally, researchers who study gesture recur to the analysis of video data. The video data are analyzed manually on the basis of predetermined coding schemes, relying on such annotation tools as ANVIL (Kipp, 2001) or ELAN (Wittenburg, Brugman, Russel, Klassmann, & Sloetjes, 2006). It has recently become possible to employ more automatic ways to analyze multimodal data. The description of movement can now be carried out using motion capture, which is a technology allowing an automatic extraction and characterization of movement parameters (e.g., space, trajectory, distance, velocity). A host of motion capture techniques are available, including the more well-known technologies, such as OptiTrack, Leap Motion, and the Microsoft Kinect. The Kinect is of particular interest due to the fact that it is inexpensive, portable, and markerless, which increases ecological validity while providing accurate depth sensing (Wasenmüller & Stricker, 2017). The Kinect is a sensor consisting of two cameras (i.e., infrared and depth) that track human skeletons in space, rendering a three-dimensional structure of movement based on joint positions (Trujillo, Simanova, Bekkering, & Özyürek, 2018).

Since its release, the Kinect has been tested and applied to a multitude of research fields, including medical (Clark et al., 2015; Galna et al., 2014), robotics (Hussein, Ali, Elmisery, & Mostafa, 2014), augmented reality (Bostanci, Kanwal, & Clark, 2015), and multimodality of communication (Trujillo et al., 2018). Being a low-cost and noninvasive motion tracking system, the Kinect could indeed be applied to the study of gesture more widely. Although the Kinect cannot fully replace manual coding, it can advance the analysis of movement in several ways. First, manual coding is extremely time-consuming, and requires more than one coder in order to calculate intercoder reliability. A substantial amount of time is spent on training the coders as well as on carrying out the actual gesture coding. Time spent on coding can be reduced by allowing motion-capture data to provide a first pass of the data, identifying individual gesture units on which the manual coders can perform further analysis. Intercoder reliability would also be increased, as motion-capture data provides an objective demarcation of the gestural units, allowing the coders to work from the same framework. Second, the manual analysis is constrained by the reliance on two-dimensional video data whereas the Kinect captures movement in three-dimensional space. This can be especially advantage when analyzing complex movements, such as pantomimes. Third, the Kinect provides the opportunity to analyze movement quantitatively, which, depending on the research question(s), can be combined with a qualitative or categorical approach to gesture coding.

Here, we provide a kinematic feature extraction protocol (with code) that quantifies several kinematic aspects of movements. We selected kinematic features in which researchers have shown interest in previous studies, and which we believe can be quantified for a variety of gestures or acts, including complex pantomimes. Because the code is available as open-source, it will additionally be possible to build off of our framework to add features that are of interest to the specific studies in which it is used.

Studies in the action and gesture domains have consistently noted the importance of size (Brand, Baldwin, & Ashburn, 2002; Campisi & Özyürek, 2013; Gerwing & Bavelas, 2004), punctuality (Brand et al., 2002), the use of holds (Gullberg & Kita, 2009), and the velocity of movements (Manera, Becchio, Cavallo, Sartori, & Castiello, 2011; Sartori, Becchio, Bara, & Castiello, 2009). We operationalize size here as being a cumulative utilization of space, and therefore include a measure of *distance*, which quantifies the accumulated distance traveled by the hands during the analyzed act. This feature will therefore capture both larger movements as well as the accumulation of many smaller movements. Punctuality was previously defined as having movements that are well marked in their beginning and end, a feature that is thought to help clearly segment the overall act for an observer (Brand et al., 2002). This fits well with work on motor control that shows that movements tend to be organized into smaller submovements. These are apparent as sharp changes in velocity, which result from changes in trajectory (e.g., reaching to grasp an object may consist of at least two submovements: an initial movement toward the object, and an additional corrective movement to ensure the hand is correctly aligned to grasp it; see, e.g., the work by Meyer, Abrams, Kornblum, Wright, & Smith, 1988). More punctual movements may therefore be seen as having more clearly defined submovements. This feature can also be seen as analogous to the gestural stroke (Kendon, 2004), allowing one to quantify the number of strokes produced. We operationalized the feature as *submovements*, which captures the number of submovements, or strokes, performed with each hand during a given act, as well as two *hold* features. Holds were defined as moments in which the hands and arms were completely still, representing a pause between submovements. These can also be seen as analogous to Kendon’s pre- or post-stroke holds. Our code calculates both *hold time* (defined as the total amount of holding time in an act) as well as *hold count* (the number of individual holds performed). Although holds can be seen as quantifying the punctuality of an act, submovements and holds can together help to identify the key movement phases, as defined by Kita and colleagues (Kita, van Gijn, & van der Hulst, 1998), that are often studied by gesture researchers. Velocity has recently been shown in several studies as important in understanding different intention underlying an act (Peeters, Holler, & Hagoort, 2013; Sartori et al., 2009). We include *peak* velocity of each hand in order to capture the fastest recorded velocity during an act. This will quantify only the fastest movement, and therefore would capture fast preparatory movements while being insensitive to holds or the inclusion of slower movements later in the act. The height at which a gesture is performed has long been of interest for gesture researchers (Gullberg & Kita, 2009; McNeill, 1994). We therefore include a measure of *vertical amplitude*, which quantifies the peak height of the hands in relation to the body of the gesturer.

In addition to presenting code for quantifying these features, we validate these new methods with respect to the established methods to provide a proof of concept. Some recent work has shown that Kinect tracking is a valid alternative to optical tracking (Fernández-Baena, Susín, & Lligadas, 2012) for clinical sciences (see Da Gama, Fallavollita, Teichrieb, & Navab, 2015, for a review), as well as several projects developing gesture recognition algorithms for the Kinect (Biswas & Basu, 2011; Paraskevopoulos, Spyrou, & Sgouropoulos, 2016). We therefore compare the kinematic analysis of gestures carried out using the our script and Kinect data with the results obtained from manually coding the same kinematic gesture features in the ELAN annotation tool.

In sum, the following paragraphs address two primary goals: (1) to provide a basic kinematic feature extraction code that can be used with Kinect, providing a platform for developing more extensive feature extraction protocols, and (2) to contrast the automatic feature analysis (Kinect) described in Trujillo et al. (2018) with the manual analysis (human coders) of gestures by means of seeing whether and to what extent the two methods, the automatic and the manual, correlate.

## Feature extraction method

### Platform

MATLAB 2015a (The MathWorks, Inc., Natick, Massachusetts, United States) was used to develop all scripts. Files saved in the C3D file format are converted to text format, after which the script imports the data and proceeds with the data processing and feature extraction.

**Fig. 1:**
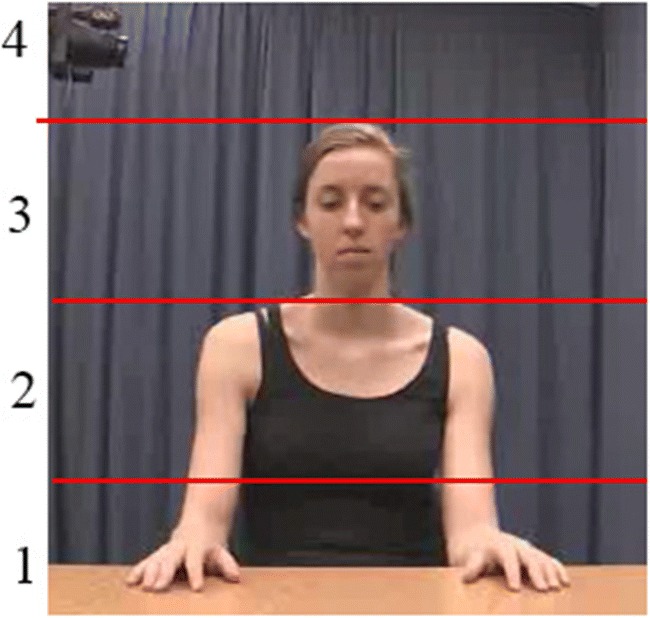
Visual representation of the vertical amplitude feature, as calculated in reference to a participant’s skeleton using the Kinect. Red lines indicate the cutoff points (approximated for illustration), with the numbers on the left indicating the value assigned to the space between the upper and lower lines. Note that 1 is bounded by the table, whereas 4 has no upper bound, and is therefore bounded by the participant’s maximum arm extension.

**Fig. 2:**
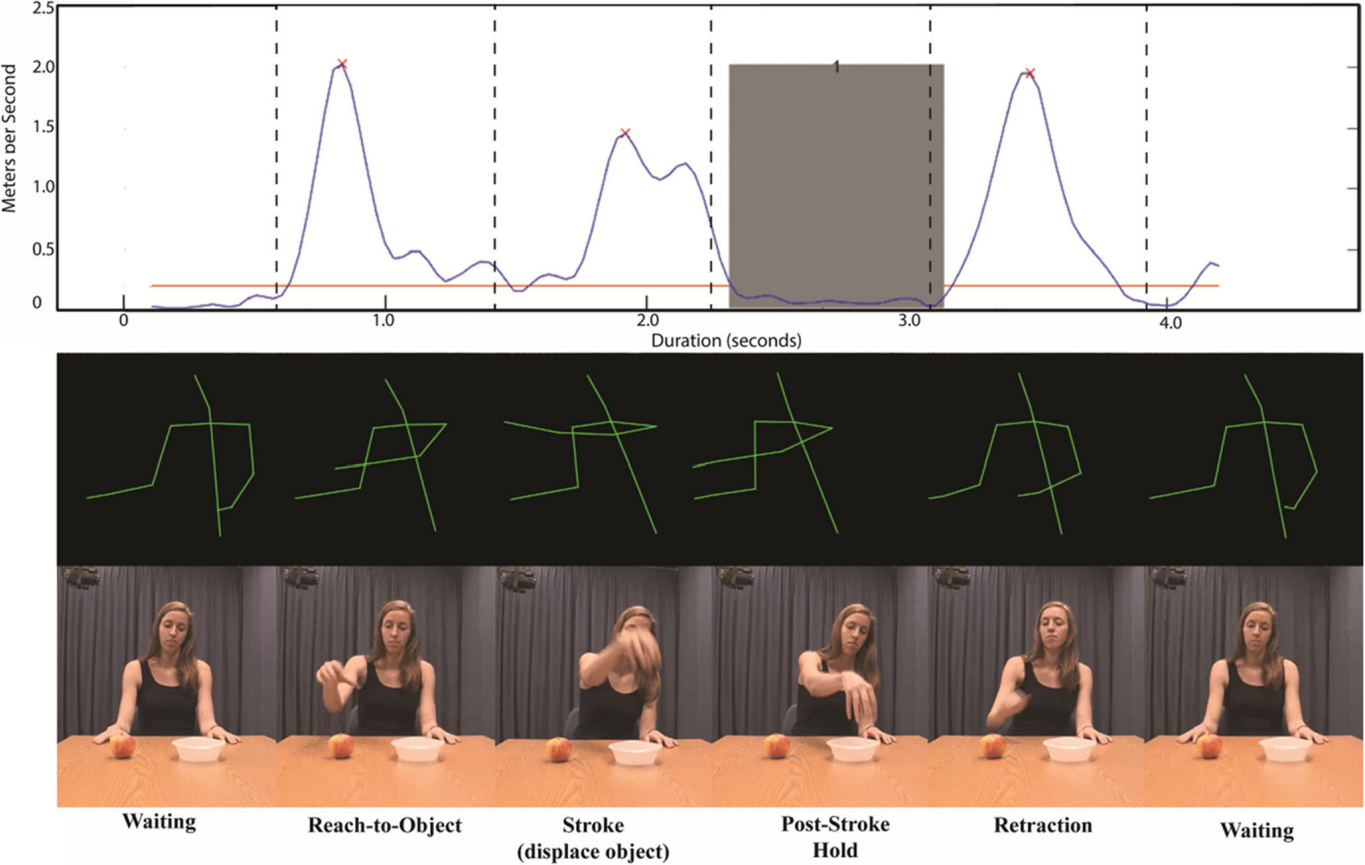
Graphical comparison of velocity profiles (data collected with Microsoft Kinect) generated by the protocol, with the corresponding video data. The depicted gesture was produced under the instruction to “place the apple in the bowl.” The *upper plot* depicts an actual output image generated by the protocol, with the addition of vertical dashed lines, which are included to show the match between the kinematic and video data. The *y*-axis depicts velocity in meters per second, whereas the *x*-axis depicts time in seconds. The horizontal red bar is the cutoff used to separate submovements from other movement noise (either measurement error or slow, nonmeaningful movements). The gray rectangle denotes a single hold, with the number printed between the bars indicating the number, or index, of the hold (e.g., if there are four separate holds in a dataset, then they will be numbered 1–4). The red Xs indicate the peak of each counted submovement. The *middle plot* shows a series of still frames, depicting the primary movement phases of the gesture as captured by the Kinect. To match the corresponding video frames, the lines only depict the torso, arms, and head. The *lower plot* shows a series of still frames, depicting the same phases as seen in the corresponding video. Below, a label is given for each depicted movement phase.

Figure 1 provides a graphical representation of how the vertical amplitude feature is calculated against the producer’s body. Figure 2 provides an example of the visualization output from the protocol, matched to corresponding video frames from the same gesture.

### Data processing

Taking the raw data, all points are smoothed using a Savitsky–Golay filter with a span of 15 and a degree of 5. This accounts for the typical jitter and motion artifacts that can occur in raw Kinect data. If available, the data will be segmented into individual acts. This step requires the user to provide an additional input, with onsets and offsets for each act. If this input is given, the output file will provide kinematic feature data for each individual act. If no onset/offset information is provided, the data file is treated as one act, and only one value for each feature is calculated (e.g., the total number of holds in the data file).

### Kinematic features

*Vertical amplitude* was defined as the highest point, in relation to a participant’s body, reached with the right dominant hand during an act. Vertical amplitude was divided into four different categories, from the lowest—which was denoted by the hand not reaching above the midline of the torso—to the highest—above the top of the head. This was calculated by comparing the hands to the spine, neck, and head in each frame of the recording (Fig. 1).

*Peak velocity* was defined as the fastest movement, reached with the right dominant hand. This was given as an absolute value in meters per second in our previous study (Trujillo et al., 2018), but was binned into seven categories by placing all peak velocity values in the present data set onto a spectrum and subsequently dividing them into seven bins, evenly distributed across the included data.

*Submovements* were defined as smaller movement segments, which were made throughout the representational gesture item. This feature is based on the work of Meyer et al. (1988), who described submovements as the individual ballistic movements that make up a given action. In shorts, each item was divided into a number of basic movements, characterized by an initial increase in velocity followed by a decrease in velocity at the points of connection of the movement segments. Submovements can be comparable to gesture strokes, which are the most semantically meaningful gesture part (Kita et al., 1998). Submovements were operationalized exceeding a velocity threshold of 0.2 m^2^, with the beginning and end marked by either the crossing of a near-zero velocity threshold (i.e., changing from static to moving) or showing a reversal from deceleration to acceleration. We used a standard peak analysis to determine the total number of peaks within the velocity profile of each hand that were at least 8 frames from the next nearest peak and with a minimum height of 0.2 m.

*Hold counts* were defined as an absence of movement in both arms and hands, for at least 300 ms. This number was utilized in Trujillo et al. (2018), due to it being the approximate minimum time length that naïve observers consistently identify as a cessation of movement. This was operationalized as sets of frames in which the hand, thumb, elbow, and shoulder of both arms all show less than 0.01 m of movement for at least 300 ms (i.e., a minimum of nine consecutive frames).

### Output

The code generates a .mat file containing all of the calculated kinematic features, with individual acts or moments separated per row in the table. If the data are not segmented by acts (see the Data Processing section above), then the one row is a summary of the data file. Additionally, a .fig plot is generated, one for each act, of the velocity profile of each hand, with submovements and holds indicated. For example of such a plot, see the top plot in Fig. 2. This plot can be useful in providing a visualization of the collected data and calculated features, but it can also be used to help guide the coding of gesture phases for further analysis. Using the save_skeleton.mat file, an additional video file can be generated of any act. This video has a black background with green lines that depict the connections between each of the measured joints. Example frames from such a file can be seen in the middle plot of Fig. 2. These “skeleton videos” can be used together with the standard recorded video to provide additional viewing angles to assist gesture coding, or as experimental stimuli. These implementations are further discussed below, in the section titled Applications.

## Validation method

### Materials

The materials in the present study consisted of a subset of videos from a production experiment from the Trujillo et al. (2018) study, in which 3-D joint tracking data were collected by employing the Microsoft Kinect V2. Although the data was collected from all 25 joints of the human body that the Kinect’s sensor is able to capture, the hips and legs were not used for any analysis. Data was collected at 30 frames per second (fps). Film data was collected at 25 fps by a camera hanging at approximately eye level, directly in front of the participant. In the Trujillo et al. (2018) study, the kinematic features that were calculated were the following: distance, vertical amplitude, peak velocity, submovements, hold time, and hold count. In the present study, we chose to analyze and compare across the two methods four kinematic features: vertical amplitude, peak velocity, submovements, and hold counts. The rationale for selecting these particular kinematic features was that they were the most amenable to hand-coding, in that it was possible to create meaningful categories for each of these features that could be captured with a naked eye. The video data used for the analysis contained only representational gestures, suggesting no videos showing actions were used for annotations. Manual data coding was carried out in the video annotation software ELAN (www.lat-mpi.eu/tools/elan/). The initial set of videos contained 120 video clips that were annotated by two human coders; however, due to data loss in the Kinect, the comparison between the manual and automatic coding is based on 111 videos.

### Validation procedure

First, Coder 1 annotated 111 videos by marking the four kinematic features in each video for each representational gesture (i.e., item). Descriptions of how the coder defined each feature are given below. Second, Coder 2, who first received training on how to code the data from Coder 1, annotated the same 111 videos. During the coding process, both coders were naïve to the kinematic values extracted by our script.

#### Manual coding of kinematic features

As with the scripted analysis, *vertical amplitude* was calculated by comparing the hands to the spine, neck and head at each frame of the recording, using the same categories as the automatic coding.

The manual coders assigned *peak velocity* values to different velocities in the range between 1 and 7. This was done after first viewing all of the videos and finding the peak movement, and then annotating each video as belong to one of the seven categories. A value of 1 therefore indicated that the fastest movement in the act was among the slowest in the dataset, whereas a value of 7 represented a movement that was among the fastest in the dataset.

For the manual coders, *submovements* were defined as the number of movements that could be segmented on the basis of an observable transition from deceleration to acceleration.

The coders defined *holds* as pauses in movement during which both hands were still in a clearly distinguishable manner for at least 300 ms.

#### Statistical comparison of coding

The analyses consisted of two steps. The first step assumed calculating Spearman’s *rho* in order to see the degree of association between the two human coders for each kinematic feature, and assessing intercoder reliability for two features in particular. That is, Cohen’s kappa was computed for *vertical amplitude* and *peak velocity* only because these features were quantified on set scales. Given that submovements and *hold counts* could take on any value of 0 or greater, assessing intercoder reliability was not possible.

The second step included comparing the Kinect features with the manual coding of Coder 1 (the second author) for which Spearman’s *rho* was used in order to determine whether the two methods were correlated. Throughout the results section, corrected *p values* are reported (Bonferroni correction was applied).

## Validation results

### Human coders

For *vertical amplitude*, the correlation was *r*_*s*_(111) = .82, *p* < .001, whereas for *submovements* it was *r*_*s*_(111) = .74, *p* < .001. *Peak velocity* and *hold counts* produced correlations of *r*_*s*_(111) = .70, *p* < .001, and *r*_*s*_(111) = .60, *p* < .001, respectively. The intercoder reliability for *vertical amplitude* was *κ* = .63, whereas that for *peak velocity* was *κ* = .40. For an overview of all results, see Supplementary Tables 1–4.

### Manual–automatic coding

*Vertical amplitude* and *submovements* produced correlations of *r*_*s*_(111) = .83, *p* < .001, and *r*_*s*_(111) = .41, *p* < .001, whereas the correlations for *peak velocity* and *hold counts* were *r*_*s*_(111) = .114, *p* = .233, and *r*_*s*_(111) = .33, *p* < .001, respectively.

## Discussion

The kinematic feature extraction toolkit presented here can be used to quantify spatial and temporal features of meaningful movements, including complex pantomimes. Together with markerless tracking technology such as the Microsoft Kinect, it provides a valuable tool for quantifying kinematic features that are important for research in the production of communicative manual acts.

To validate this method, we compared automatically extracted kinematic features, based on Kinect data, with manually coded kinematic features, based on video data. The results of this validation process show that the Kinect can robustly measure both spatial and temporal kinematics of pantomimes, with automatically extracted features (i.e., *vertical amplitude*, *submovements*, and *hold counts*) largely similar to the manually coded features. Although the *peak velocity* showed very poor overlap between the manual and automatic codings, intercoder reliability in the manual coding for this feature was also lower. This suggests that the proposed method of automatic extraction may measure this feature more robustly.

### Human coders

The gesture coding between two manual coders resulted in high correlations for the kinematic features of *vertical amplitude*, *submovements*, and *peak velocity*, whereas the correlation for *hold count* was slightly lower than those for the other three features. Although coding of peak velocity was highly correlated between the coders, there was somewhat lower reliability, as indicated by the lower kappa score. This suggests that although the manual coders were consistent in ranking the videos (i.e., providing larger numbers for videos with faster movements), there was less reliability for selecting the exact same category. Due to the more subjective nature of this feature, it is not surprising that reliability was somewhat lower. However, the overall high correlations between coders indicate that the coding of these features was carried out in a consistent and replicable manner.

### Manual–automatic coding

Overall, good agreement was seen in *vertical amplitude* and the number of *submovements*. Because *vertical amplitude* was relatively straightforward to define, with a clear reference point (participant body) against which to compare the height of the hands, this result was very much expected. *Submovements* also showed high overlap. The high correlation between human and automatic coding suggests that our automatic approach captures individual submovements, at least on the coarse level at which a human observer may also segment an act into individual movements. This is important, because it shows that the automatic coding captures the primary movement boundaries in a similar way to human coders. Since submovements can be seen as analogous to gesture strokes, this provides some validation of the process as an objective and automatic way to code these gesture units.

When coding *hold counts*, we found a significant positive correlation, although the fit of the model was lower than that for *vertical amplitude* or *submovements*. Closer inspection of the data revealed that in some cases it was difficult for the manual coders to accurately delineate the beginning and end of individual holds, due to the presence of small movements or a series of very brief holds. In this case, we suggest that the holds are likely to be more accurately counted by the automatic approach, as there are clear cutoff points for movement and duration.

Although *peak velocity* did not show a strong correspondence between automatic and manual coding, we suggest that this may have been due to differences in which movements were coded as being the fastest. When qualitatively comparing the automatic and manual analyses, it was noticed that manual coders would reliably capture larger movement segments within a given gesture but fail to extract very fast but short movements. The association between the two methods for *peak velocity* relied on the assumption that overall the same submovements were extracted by the Kinect and the human coder, which generally was true, however, when this was not the case, the fastest submovement recorded by the Kinect would be a different submovement labeled as the fastest by the human coder. In other words, the outcome of movement segmentation mattered for both the *submovements* and *peak velocity*. These results suggest that velocity is a very difficult metric to code visually due to its being mathematically very precise, and therefore it may be made more accessible by using more robust measuring devices, such as the Kinect. In sum, the somewhat lower overlap between the automatic and manual methods for *peak velocity* and *hold counts* does not undermine the robustness of the obtained results. On the contrary, this indicates that the Kinect can be an effective means to code kinematic features that provide significant challenges for accurate manual coding. Using a mathematical approach with strict criteria therefore allows fine-grained and accurate quantification of these features.

### Implementation

**Fig. 3:**
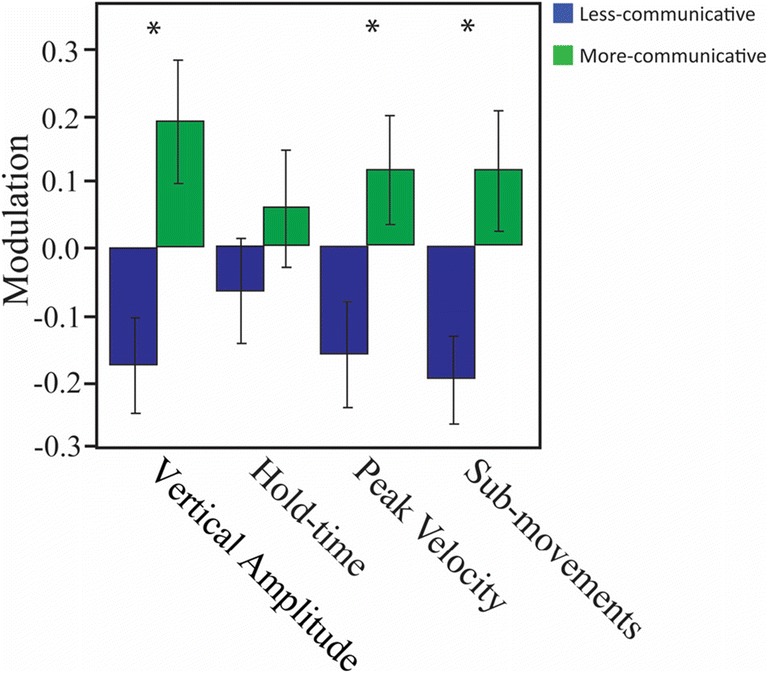
Kinematic modulation data in more and less communicative gestures, reproduced with permission from the data of Trujillo et al. (2018). Kinematic features are displayed along the *x*-axis, whereas modulation values (deviation from sample mean) are displayed along the *y*-axis. Blue bars depict the less communicative context, whereas green bars depict the more communicative context. ^*^p < .001.

Our approach was recently applied in a study by Trujillo et al. (2018), in which participants performed 31 object-directed actions (e.g., brushing hair, folding a paper, etc.) and the corresponding representational gestures (i.e., enacting the same actions without the object being present) in two settings. The difference between these two settings was that in the first setting, participants were induced to believe that someone was observing their actions and gestures with the aim to learn from them (i.e., more communicative context), whereas in the second setting, although they again believed they were being observed, the participants assumed they were performing the actions and gestures for themselves (i.e., less communicative context). The key finding of the production experiment in Trujillo et al.’ (2018) study was that both actions and gestures were kinematically modulated with respect to the context in which they were performed, with submovements and vertical amplitude being increased in both actions and gestures in the more as compared to the less communicative context. Peak velocity was additionally increased in more as compared to less communicative gestures (Fig. 3). The comprehension experiment in the same study showed that the kinematic modulations of gestures were reliably perceived and utilized by the addressees, in that naïve observers used the increased vertical amplitude to infer whether actors were performing the gesture for themselves or for the viewer. A follow-up study using the same production data additionally showed that these increases in submovements, peak velocity, and holds improve comprehension of the semantic content of the act (Trujillo, Simanova, Bekkering, & Özyürek, submitted for publication). Together, these findings show that our toolkit can quantify kinematic features that are important characteristics of the communicative context of a manual act, and that these same features are used by addressees to understand intention and semantic content.

### Limitations

Although this validation study shows promise for the quantification of kinematic features in action and gesture research, it should be noted that the features extracted and validated here only measure the qualities of movement in a given act. We therefore do not expect this methodology to replace manual coding, particularly in the case of qualitative classification of gestures. The feature extraction is also meant to capture a type of summary information of a given manual act. That is to say, this does not generate online or continuous coding of all movement, but is meant to be applied to a single act or set of movements that one wishes to characterize. Although the present protocol utilizes predefined start and end points to define what constitutes a single act or the time frame of analysis, this could be modified to be used together with automatic segmentation- or gesture-defining tools (see, e.g., the work by De Beugher, Brône, & Goedemé, 2018).

### Applications

Using the Microsoft Kinect to capture gesture production and automatically extract kinematic features can be an important tool for researchers interested in meaningful movements. Previous research has shown that velocity of pointing gestures may be modulated by the communicative context in which they are performed (Peeters et al., 2013), and the size (Campisi & Özyürek, 2013; Bavelas, Gerwing, Sutton, & Prevost, 2008) or height (Hilliard & Cook, 2016) of gestures may also be modulated by a common ground in knowledge between the speaker and addressee. Furthermore, velocity and size of communicative gestures has also been shown to affect the response of interactional partners (Innocenti, de Stefani, Bernardi, Campione, & Gentilucci, 2012), as well as to signal communicative intention (Trujillo et al., 2018b) and clarify the semantics of the act (Trujillo et al., 2018a). Studies on communicative actions may also benefit from this tool. When compared to interacting with other adults, child-directed (Brand et al., 2002) as well as robot-directed actions (Vollmer et al., 2009) are modulated by distinct kinematic features. Similar features may also be useful in differentiating between various adult interactive contexts, such as demonstration and joint action coordination (McEllin, Knoblich, & Sebanz, 2018). Clinicians may also benefit from such analysis, as pantomime production is often used when assessing aphasia (Goldenberg, Hermsdörfer, Glindemann, Rorden, & Karnath, 2007; Hermsdörfer, Li, Randerath, Goldenberg, & Johannsen, 2012). An additional advantage to this approach is that the Kinect does not require reflective markers or other physical components attached to the participant, allowing a somewhat more ecological approach in which the participant may be less aware of the fact that their movements are being recorded. In the case of clinical applications, this markerless aspect allows the tool to be implemented without providing any additional discomfort to the patient.

**Fig. 4:**
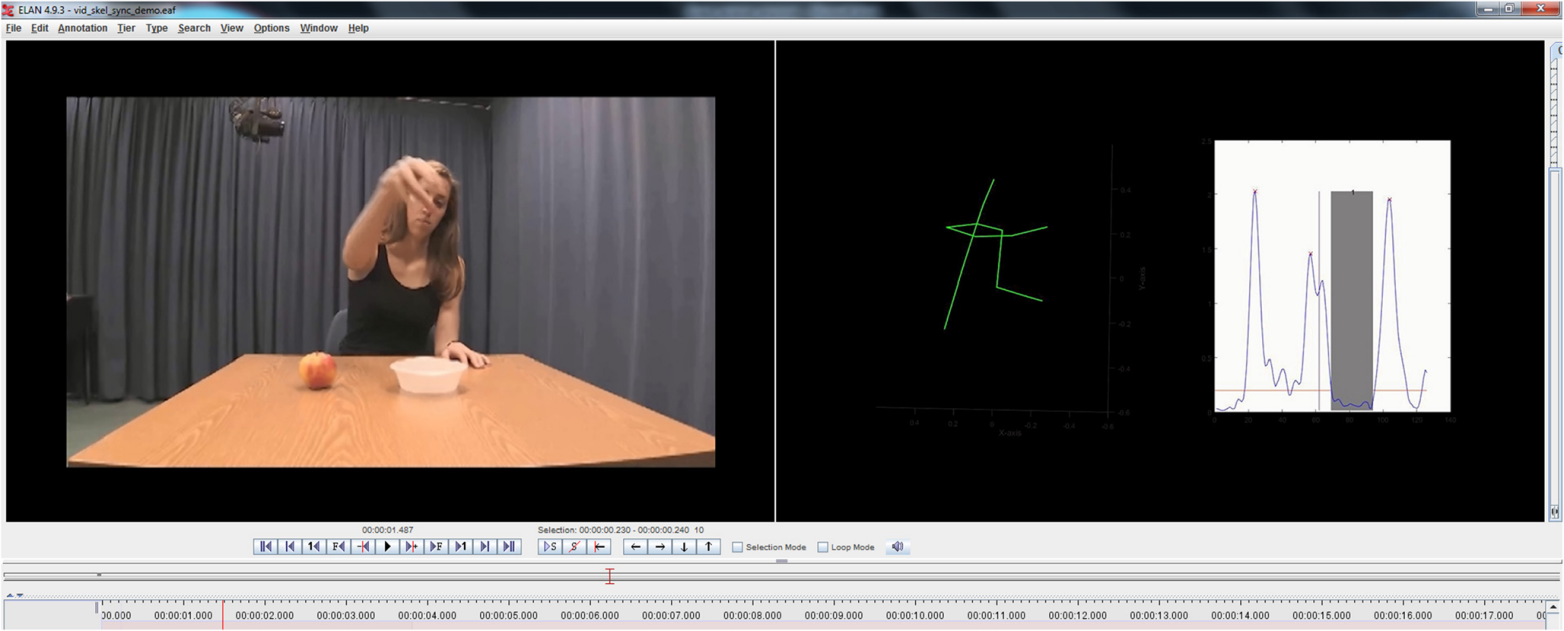
Example of a video and kinematic pairing in ELAN. On the left, the standard video recording is being played, while on the right a skeleton of the motion capture data as well as the velocity profile of the right hand are being played simultaneously. Note the vertical bar on the velocity profile, which moves from left to right as the video plays, allowing a coder to see to which part of the plot the current video frame corresponds.

Aside from the direct quantification of specific features, the velocity profile that is provided as output (see Fig. 2) can also be used side by side with video data in order to assist in the manual coding of strokes and holds. Although the gestural units themselves are accurately defined in time by the Kinect code, a manual coder can more easily code the qualitative or categorical features of these units. For example, by finding the onset of a velocity peak that has been marked as a submovement by the toolkit, one can easily and precisely find the onsets (and offsets) of strokes. Similarly, the onsets and offsets of holds are made more precise by finding the onsets and offsets as defined by the toolkit. In Fig. 4, we give an example of a video paired with a Kinect-acquired velocity profile video that can be used to find the onsets and offsets of relevant gesture phases.

Finally, Kinect data can be used to supplement video data, thanks to the Kinect data’s three-dimensional nature. Although gesture data in the lab are often acquired with multiple cameras capturing distinct angles, fieldwork may make such multicamera setups more difficult. In this case, standard video data may be used as the primary source for coding data, but the Kinect acquisition would additionally provide a velocity profile output to support the coding of gesture phases, as well as any number of angles of visualization to reduce ambiguities that may come from typical 2-D data and limited angles of acquisition. As an example of this, Fig. 4 depicts the Kinect acquisition playing alongside a video recording, where the movements can be seen from a slightly rotated viewing angle that is provided.

## Summary

Our novel kinematic feature extraction protocol provides a robust measure of spatial and temporal kinematics, with extracted features being representative of what human observers can reliably code, while additionally allowing access to features that human coders have difficulty quantifying. Overall, we believe this methodology can be a useful tool for gesture researchers, clinicians, and others interested in quantifying the kinematics of meaningful human movement.

## Electronic supplementary material

ESM 1 (DOCX 16 kb)

ESM 2 (7Z 21 kb)

ESM 3 (7Z 6207 kb)

ESM 4 (SAV 5 kb)

## References

[CR1] Anzulewicz A, Sobota K, Delafield-Butt JT (2016). Toward the autism motor signature: Gesture patterns during smart tablet gameplay identify children with autism. Scientific Reports.

[CR2] Bavelas J, Gerwing J, Sutton C, Prevost D (2008). Gesturing on the telephone: Independent effects of dialogue and visibility. Journal of Memory and Language.

[CR3] Biswas K. K., & Basu, S. K. (2011). Gesture recognition using Microsoft Kinect®. In *Proccedings of the 5th International Conference on Automation, Robotics and Applications* (pp. 100–103). Piscataway, NJ: IEEE Press.

[CR4] Bostanci E, Kanwal N, Clark AF (2015). Augmented reality applications for cultural heritage using Kinect. Human-Centric Computing and Information Sciences.

[CR5] Brand RJ, Baldwin DA, Ashburn LA (2002). Evidence for “motionese”: Modifications in mothers’ infant-directed action. Developmental Science.

[CR6] Campisi E, Özyürek A (2013). Iconicity as a communicative strategy: Recipient design in multimodal demonstrations for adults and children. Journal of Pragmatics.

[CR7] Church RB, Alibali MW, Kelly SD (2017). *Why gesture? How the hands function in speaking, thinking and communicating*.

[CR8] Clark RA, Vernon S, Mentiplay BF, Miller KJ, McGinley JL, Pua Y, Bower KJ (2015). Instrumenting gait assessment using the Kinect in people living with stroke: Reliability and association with balance tests. Journal of Neuroengineering and Rehabilitation.

[CR9] Da Gama A, Fallavollita P, Teichrieb V, Navab N (2015). Motor rehabilitation using Kinect: A systematic review. Games Health Journal.

[CR10] De Beugher S, Brône G, Goedemé T (2018). A semi-automatic annotation tool for unobtrusive gesture analysis. Language Resources and Evaluation.

[CR11] Fernández-Baena, A., Susín, A., & Lligadas, X. (2012). Biomechanical validation of upper-body and lower-body joint movements of Kinect motion capture data for rehabilitation treatments. In F. Xhafa, L. Barolli, F. Pop, X. Chen, & V. Cristea (Eds.), *2012 Fourth International Conference on Intelligent Networking and Collaborative Systems* (pp. 656–661). Piscataway, NJ: IEEE Press.

[CR12] Galna B, Barry G, Jackson D, Mhiripiri D, Olivier P, Rochester L (2014). Accuracy of the Microsoft Kinect sensor for measuring movement in people with Parkinson’s disease. Gait and Posture.

[CR13] Gerwing J, Bavelas J (2004). Linguistic influences on gesture’s form. Gesture.

[CR14] Goldenberg G, Hartmann K, Schlott I (2003). Defective pantomime of object use in left brain damage: Apraxia or asymbolia?. Neuropsychologia.

[CR15] Goldenberg G, Hermsdörfer J, Glindemann R, Rorden C, Karnath H-O (2007). Pantomime of tool use depends on integrity of left inferior frontal cortex. Cerebral Cortex.

[CR16] Gonzalez Rothi LJ, Heilman KM, Watson RT (1985). Pantomime comprehension and ideomotor apraxia. Journal of Neurology, Neurosurgery, and Psychiatry.

[CR17] Gullberg M, Kita S (2009). Attention to speech-accompanying gestures: Eye movements and information uptake. Journal of Nonverbal Behavior.

[CR18] Hermsdörfer J, Li Y, Randerath J, Goldenberg G, Johannsen L (2012). Tool use without a tool: Kinematic characteristics of pantomiming as compared to actual use and the effect of brain damage. Experimental Brain Research.

[CR19] Hilliard C, Cook SW (2016). Bridging gaps in common ground: Speakers design their gestures for their listeners. Journal of Experimental Psychology: Learning, Memory, and Cognition.

[CR20] Humphries S, Holler J, Crawford TJ, Herrera E, Poliakoff E (2016). A third-person perspective on co-speech action gestures in Parkinson’s disease. Cortex.

[CR21] Hussein MA, Ali AS, Elmisery FA, Mostafa R (2014). Motion control of robot by using Kinect sensor. Research Journal of Applied Sciences, Engineering and Technology.

[CR22] Innocenti A, de Stefani E, Bernardi NF, Campione GC, Gentilucci M (2012). Gaze direction and request gesture in social interactions. PLoS ONE.

[CR23] Kendon A (2004). *Gesture: Visible actions as utterance*.

[CR24] Kipp, M. (2001). ANVIL: A generic annotation tool for multimodal dialogue. In P. Dalsgaard, B. Lindberg, H. Benner, & Z.-H. Tan (Eds.), *Proceedings of Eurospeech 2001* (pp. 1367–1370). Washington, DC: ISCA. Retrieved from dblp.uni-trier.de/db/conf/interspeech/interspeech2001.html

[CR25] Kita, S., van Gijn, I., & van der Hulst, H. (1998). Movement phases in signs and co-speech gestures, and their transcription by human coders. In I. Wachsmuth & M. Fröhlich (Eds.), *Gesture and Sign Language in Human–Computer Interaction: GW 1997*. *Proceedings of the International Gesture Workshop* (pp. 23–35). Berlin, Germany: Springer.

[CR26] Manera V, Becchio C, Cavallo A, Sartori L, Castiello U (2011). Cooperation or competition? Discriminating between social intentions by observing prehensile movements. Experimental Brain Research.

[CR27] McEllin L, Knoblich G, Sebanz N (2018). Distinct kinematic markers of demonstration and joint action coordination? Evidence from virtual xylophone playing. Journal of Experimental Psychology: Human Perception and Performance.

[CR28] McNeill D (1994). *Hand and mind: What gestures reveal about thought*.

[CR29] Meyer DE, Abrams RA, Kornblum S, Wright CE, Smith JE (1988). Optimality in human motor performance: Ideal control of rapid aimed movements. Psychological Review.

[CR30] Paraskevopoulos G, Spyrou E, Sgouropoulos D (2016). A real-time approach for gesture recognition using the Kinect sensor. *Proceedings of the 9th Hellenic Conference on Artificial Intelligence—SETN ’16*.

[CR31] Peeters D, Holler J, Hagoort P, Knauff M, Pauen M, Sebanz N, Wachsmuth I (2013). Getting to the point: The influence of communicative intent on the kinematics of pointing gestures. *Cooperative minds: Social interaction and group dynamics*. *Proceedings of the 35th Annual Meeting of the Cognitive Science Society*.

[CR32] Sartori L, Becchio C, Bara BG, Castiello U (2009). Does the intention to communicate affect action kinematics?. Consciousness and Cognition.

[CR33] Trujillo JP, Simanova I, Bekkering H, Özyürek A (2018). Communicative intent modulates production and perception of actions and gestures: A Kinect study. Cognition.

[CR34] Vollmer, A.-L., Lohan, K. S., Fischer, K., Nagai, Y., Pitsch, K., Fritsch, J., . . . Wredek, B. (2009). People modify their tutoring behavior in robot-directed interaction for action learning. In *2009 IEEE 8th International Conference on Development and Learning* (pp. 1–6). Piscataway, NJ: IEEE Press. 10.1109/DEVLRN.2009.5175516

[CR35] Wasenmüller, O., & Stricker, D. (2017). Comparison of Kinect V1 and V2 depth images in terms of accuracy and precision. In C.-S. Chen, J. Lu, K.-K. Ma (Eds.), *Computer Vision—ACCV 2016 Workshops* (Part II, pp. 34–45). Cham, Switzerland: Springer.

[CR36] Wittenburg, P., Brugman, H., Russel, A., Klassmann, A., & Sloetjes, H. (2006). ELAN: A professional framework for multimodality research. In *Proceedings of the 5th International Conference on Language Resources and Evaluation* (pp. 1556–1559). Cham, Switzerland: Springer.

