# Supplementary material for: Toward the markerless and automatic analysis of kinematic features: A toolkit for gesture and movement research
Source: Behav Res Methods. 2018 Aug 24;51(2):769–77. doi: 10.3758/s13428-018-1086-8 (PMC6478643; doi:10.3758/s13428-018-1086-8)
Supplement: Supplementary file 1 — (DOCX 16 kb) [file 13428_2018_1086_MOESM1_ESM.docx]

Supplementary table 1. Inter-rater agreement for Vertical amplitude

|  | |  | Coder 2 | |  |
| --- | --- | --- | --- | --- | --- |
| Coder 1 | value | 1 | 2 | 3 | 4 |
|  | 1  2  3  4 | **9** | 19 |  |  |
|  |  | 1 | **45** | 1 |  |
|  |  |  | 5 | **23** | 1 |
|  |  |  |  |  | **7** |

Supplementary table 2. Inter-rater agreement for Hold-count

|  | |  | Coder 2 | | |  |
| --- | --- | --- | --- | --- | --- | --- |
| Coder 1 | value | 0 | 1 | 2 | 3 | 4 |
|  | 0  1  2  3  4 | **42** | 2 | 1 | 1 |  |
|  |  | 6 | **29** | 10 | 1 | 1 |
|  |  | 3 | 1 | **7** |  | 1 |
|  |  | 3 | 2 |  |  |  |
|  |  |  |  | 1 |  |  |

Supplementary table 3. Inter-rater agreement for Peak velocity

|  | |  | Coder 2 | | | |  |  |
| --- | --- | --- | --- | --- | --- | --- | --- | --- |
| Coder 1 | value | 2 | 3 | 4 | 5 | 6 | 7 | 8 |
|  | 2  3  4  5  6  7  8 | **2** | 1 | 1 | 1 |  |  |  |
|  |  |  | **13** | 4 | 4 | 1 |  |  |
|  |  |  | 7 | **22** | 3 | 3 | 1 |  |
|  |  |  | 2 | 5 | **5** | 4 | 1 |  |
|  |  |  | 1 | 1 | 2 | **3** | 1 |  |
|  |  |  |  |  |  | 3 | **4** | 2 |
|  |  |  |  | 1 |  | 3 | 2 | **8** |

Supplementary table 4. Inter-rater agreement for Sub-movements

|  | |  |  | | | | Coder 2 | | |  |  |  |  |  |
| --- | --- | --- | --- | --- | --- | --- | --- | --- | --- | --- | --- | --- | --- | --- |
| Coder 1 | value | 2 | 3 | 4 | 5 | 6 | 7 | 8 | 9 | 10 | 11 | 12 | 13 | 15 |
|  | 3  4  5  6  7  8  9  10  11  12  13  14  17 | 1 | 5 | 1 |  |  |  | 1 |  |  |  |  |  |  |
|  |  |  | **2** | 5 |  |  |  |  |  |  |  |  |  |  |
|  |  |  |  | **8** | 7 | **3** |  |  |  |  |  |  |  |  |
|  |  |  | 1 |  | **2** | **5** | **2** |  | 1 | 1 |  |  |  |  |
|  |  | 1 | 1 | 2 | 1 | **3** | 1 | 2 | 1 | 2 |  |  |  |  |
|  |  |  |  | 1 | 1 | 2 | 1 | **6** | **3** |  |  |  |  |  |
|  |  |  |  |  | 1 | 5 | 2 | 1 | **3** |  |  |  |  |  |
|  |  |  | 1 |  |  |  |  | 1 | 1 | 1 |  |  |  |  |
|  |  |  |  |  |  |  |  | 2 | 2 | 1 | **3** |  |  |  |
|  |  |  |  |  |  |  |  | 1 |  | 2 | **2** |  |  |  |
|  |  |  |  |  |  | 1 | 1 | 1 |  |  |  |  | 1 |  |
|  |  |  |  |  |  |  |  |  |  |  |  | **3** |  |  |
|  |  |  |  |  |  |  |  |  |  |  |  |  |  | 1 |
